# Increased Relative Delta Bandpower and Delta Indices Revealed by Continuous qEEG Monitoring in a Rat Model of Ischemia-Reperfusion

**DOI:** 10.3389/fneur.2021.645138

**Published:** 2021-04-07

**Authors:** Luan Oliveira Ferreira, Bruna Gerrits Mattos, Vanessa Jóia de Mello, Arnaldo Jorge Martins-Filho, Edmar Tavares da Costa, Elizabeth Sumi Yamada, Moisés Hamoy, Dielly Catrina Favacho Lopes

**Affiliations:** ^1^Laboratory of Experimental Neuropathology, João de Barros Barreto University Hospital, Federal University of Pará, Belém, Brazil; ^2^Laboratory of Pharmacology and Toxicology of Natural Products, Institute Biological Science, Federal University of Pará, Belém, Brazil; ^3^Pathology Section, Evandro Chagas Institute, Ananindeua, Brazil

**Keywords:** quantitative electroencephalography, ischemic stroke, middle cerebral artery occlusion, delta wave, brain injury, delta/alpha ratio

## Abstract

The present study describes the electroencephalographic changes that occur during cerebral ischemia and reperfusion in animals submitted to transient focal cerebral ischemia by middle cerebral artery occlusion (MCAO) for 30 min. For this, male Wistar rats were divided into two groups (*n* = 6 animals/group): (1) sham (control) group, and (2) ischemic/reperfusion group. The quantitative electroencephalography (qEEG) was recorded during the ischemic and immediate reperfusion (acute) phases, and then once a day for 7 days after the MCAO (subacute phase). The acute phase was characterized by a marked increase in the relative delta wave band power (*p* < 0.001), with a smaller, but significant increase in the relative alpha wave bandpower in the ischemic stroke phase, in comparison with the control group (*p* = 0.0054). In the immediate reperfusion phase, however, there was an increase in the theta, alpha, and beta waves bandpower (*p* < 0.001), but no alteration in the delta waves (*p* = 0.9984), in comparison with the control group. We also observed high values in the delta/theta ratio (DTR), the delta/alpha ratio (DAR), and the (delta+theta)/(alpha+beta) ratio (DTABR) indices during the ischemia (*p* < 0.05), with a major reduction in the reperfusion phase. In the subacute phase, the activity of all the waves was lower than that of the control group (*p* < 0.05), although the DTR, DAR, and DTABR indices remained relatively high. In conclusion, early and accurate identification of decreased delta wave bandpower, DTR, DAR, and DTABR indices, and an increase in the activity of other waves in the immediate reperfusion phase may represent an important advance for the recognition of the effectiveness of reperfusion therapy.

## Introduction

Worldwide, stroke is one of the main causes of motor and functional impairment, adult-onset disability (1), and reduced quality of life (2). In the acute phase of cerebral ischemia, however it can be difficult to predict whether individuals with severe neurological deficits will recover their functions fully or partially, or whether they will die (3). Although the anatomical location and the severity of the stroke are considered to be strong predictive indices of disability, recovery is not always linked to the size or extent of the initial lesion (4), but rather to the recuperation of cerebral blood flow and synaptic plasticity between neuronal cells (5, 6).

In this context, the electrical activity of the neurons is closely linked to the flow of blood to the brain, on a space-time scale, which is often referred to as the neurovascular coupling (7). If the blood flow is interrupted, there is a dysfunction of this neurovascular coupling, which leads metabolically to an electrical imbalance in the brain (7, 8). This breakdown of homeostasis causes failure of the oxidative phosphorylation and ATP production. This affects the Na^+^/K^+^ ATPase and Ca^++^ pumps, resulting in plasma membrane depolarization and increased intracellular Ca^++^, respectively. This will activate a number of several death-signaling proteins, such as caspases, proteases, lipases, and DNAses, which will result in neuronal death (9).

Quantitative electroencephalography measures the electrical activity of the brain, primarily through the quantification of excitatory, and inhibitory postsynaptic currents in the cortical pyramidal cells layers. This approach provides data on raw electroencephalographic (EEG) signals and has been widely used as an electrophysiological tool for prognosis (5, 10, 11).

As a non-invasive technique, qEEG is considered to be an effective complementary tool for the traditional clinical evaluation of patients with altered mental capabilities, in particular, in the post-stroke period (12, 13). Distinct relative wave bandpower activity patterns are associated with specific brain functions, and can be related systematically to the varying degrees of neuron survival in the regions affected by a stroke, contributing to the prognosis (5, 14).

A growing body of evidence indicates that individuals who have suffered an ischemic or hemorrhagic stroke present a significant increase in delta wave bandpower (1–4 Hz), with either a decrease or no alteration in the bandpower of their alpha (8–12 Hz) or beta (12–28 Hz) waves (5, 13–16). These waves can reveal ionic and/or metabolic alterations that reflect ischemic brain damage (17). These changes in wave frequencies provide the basis for the calculation of the qEEG indices, i.e., the DAR, DTABR, and the DTR, which tend to be altered following a stroke (13, 16, 18).

Although these indices have proved useful for the description of the correlation between the stroke and the clinical status of the patient, very little is known about brain activity in the acute and immediate reperfusion phases. In addition, serial neuromonitoring after the return of the blood flow would be extremely important to evaluate the effectiveness of therapies based on thrombolytic or neurorepair drugs (19, 20).

Given the lack of studies on the electrical activity of the brain in the acute and reperfusion phases, which would provide important insights into the evolution of the stroke, the present study was based on the continuous neuromonitoring of cerebral ischemia and reperfusion (immediate reperfusion and a follow-up of 7 days) in animals submitted to transient focal cerebral ischemia caused by middle cerebral artery occlusion.

## Materials and Methods

### Animals

Male Wistar rats (*n* = 12) aged 10–12 weeks and weighing 300 g (± 20 g) were used in the present study. All the animals were housed in a controlled environment (22 ± 2°C; 12/12 h light/dark cycle) in standard cages (48 × 38 × 21 cm) with *ad libitum* access to food and water. The experimental procedures were approved by the relevant Brazilian federal agencies and were in accordance with the Brazilian National Council for the Control of Animal Experimentation and the Ethics Committee on Use of Animals of the Biological Sciences Institute at the Federal University of Pará (CEUA/UFPA no. 1348060318). The experimental data reported in this study were also collected in compliance with the ARRIVE guidelines (Animal Research: Reporting *in vivo* Experiments). All necessary precautions were taken to prevent animal suffering and distress.

### Experimental Design

**Figure 1:**
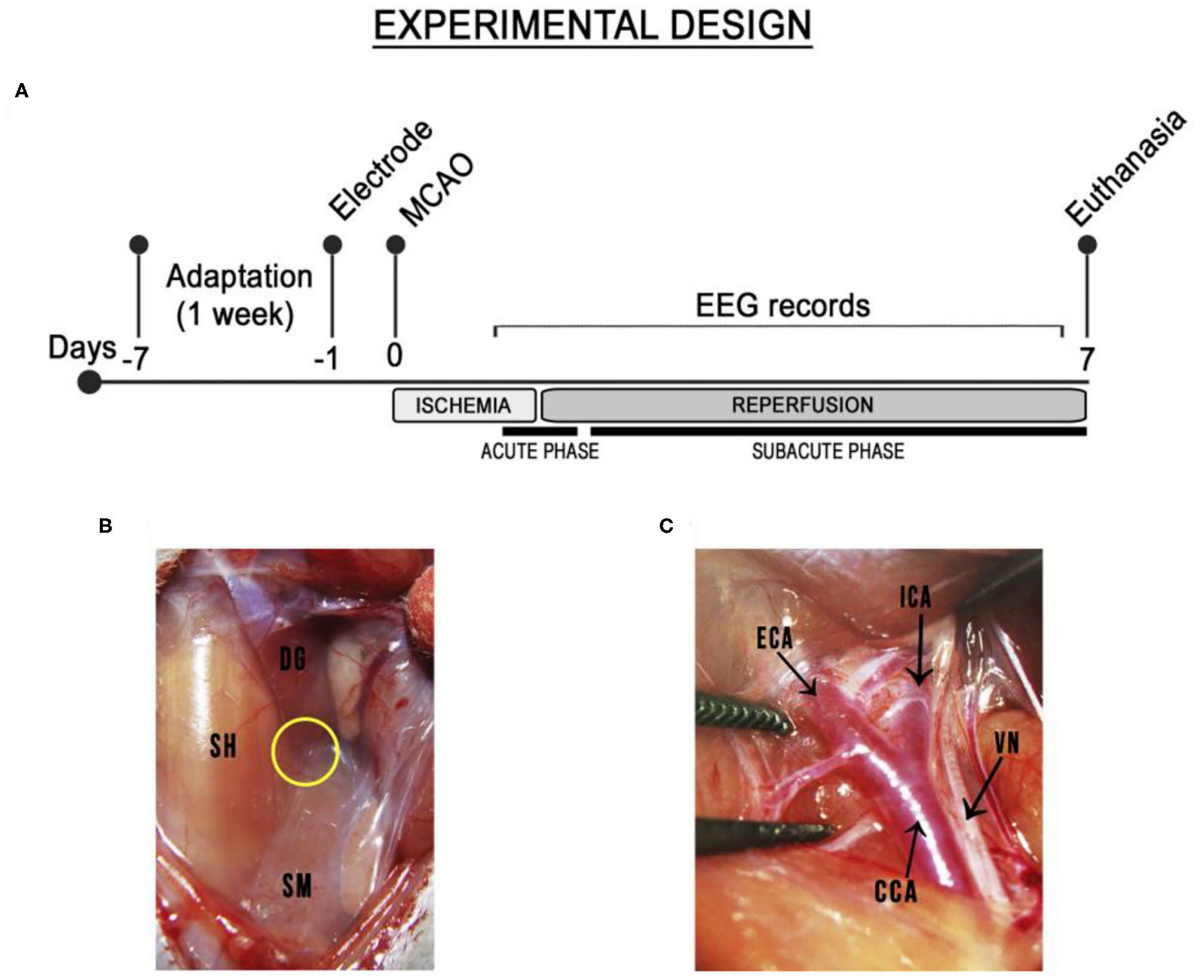
Schematic diagram of the timeline of the transient ischemic stroke and the middle cerebral artery occlusion surgery. **(A)** Experimental design. Electrode: implantation of electrodes. MCAO: middle cerebral artery occlusion. Ischemia: animals submitted to MCAO for 30 min. Reperfusion: reperfusion monitored for 7 days after the MCAO. **(B)** Insertion point among the three muscles (yellow circle): the sternohyoid (SH), medial to incision; the digastric (DG), identified by its shiny white portion), and the sternomastoid muscle (SM). **(C)** The common, external, and internal carotid arteries (CCA, ECA, ICA) were exposed and the vagus nerve (VN) was dissected carefully from the common and internal carotid arteries.

The study animals were maintained in the facility for at least 7 days for adaptation prior to the surgical procedure (Figure 1A). The EEG electrodes were implanted in the cortex 24 h prior to the MCAO surgery. The animals were subsequently divided into two groups (*n* = 6 animals/group): sham (control) and ischemic (animals submitted to MCAO for 30 min) groups. Both groups were monitored up to 7 days after reperfusion. The EEGs were recorded in the last 5 min of the MCAO (i.e., at 25–30 min after the initiation) and in the first 10 min of reperfusion, referred to here as the acute phase. Subsequently, the EEGs were recorded once per day (5 min) for 7 days after the MCAO (i.e., the subacute phase). After euthanasia, the brains were extracted, sectioned, and stained with cresyl violet (0.3%) to measure the percentage of the area of the hemisphere that had suffered infarction. All the procedures were conducted strictly between 08:00 AM and 11:00 AM.

### Middle Cerebral Artery Occlusion Surgery

The MCAO surgery was conducted as described previously, with some modifications (21). Briefly, the rats were anesthetized intraperitoneally (i.p.) with ketamine (80 mg/kg) and xylazine (10 mg/kg) and placed under a heated blanket. After the abolishment of the corneal reflex, the left common carotid artery (CCA) was exposed through an incision of the cervical midline and guided by the triangle formed by three muscles: the sternohyoid, the digastric, and the sternomastoid muscles (Figure 1B). The vagus nerve was in the lateral aspect of both common and internal carotid arteries and was carefully dissected from them. The carotid arteries were then exposed (Figure 1C) and the external carotid artery was tied off as distally. After that, microsurgical clips were placed on common and internal carotid arteries near the bifurcation and partial arteriotomy was performed on the external carotid artery to insert a silicone-coated nylon monofilament (Doccol Corp., Redlands, CA, USA) and then along the internal carotid artery (ICA) up to 20 mm distally from the carotid bifurcation, where it remained for 30 min. The sham-operated rats were anesthetized and the carotid bifurcation was exposed, but no filament was inserted. The incision was sutured and the rats were returned to their cages as soon as they regained consciousness.

### Electrocorticographic Recordings and Data Analyses

The EEGs were recorded as described by Estumano et al. (22) and Ferreira et al. (23). Briefly, the animals were anesthetized with ketamine (80 mg/kg, i.p.) and xylazine (10 mg/kg, i.p.). After the abolishment of the corneal reflex, the animal was placed in a stereotaxic apparatus for the implantation of stainless steel electrodes (exposed tip 1.0 mm in diameter), which were placed on the dura mater above the pre-frontal cortex at the Bregma coordinates −0.96 mm and ± 1.0 mm lateral and were fixed with dental acrylic cement. Following surgery, the animals were kept in individual cages. For the records, the electrodes were connected to a digital data acquisition system composed of a high impedance amplifier (Grass Technologies, P511), an oscilloscope (Protek, 6510), and a data acquisition and digitalization board (National Instruments, Austin, TX). Data were collected continuously at 1 kHz, at a low pass of 3 kHz and high pass of 0.3 Hz. During the recording sessions, the animals were confined to a restricted space in acrylic boxes (20 × 45 × 15 cm). The EEGs were recorded by a digital data acquisition system and the offline analysis was run as described by Santos et al. (24) and Hamoy et al. (25). Thus, offline analyses were run using a tool built in the Python programming language (version 2.7), with “Numpy” and “Scipy” libraries being used for the mathematical processing and a “matplolib” library to obtain the data. A graphic interface was developed using the PyQt4 library.

The data collection was divided into two phases, the acute and the subacute phases. In the acute phase, the animals were immobilized carefully for 10 min for habituation, to avoid interference, and the basal EEG activity was recorded for 10 min with the animal awake and subsequently, with the animal anesthetized (used as the control for the qEEG analyses of the ischemic stroke phase); the recordings of the acute phase were divided into the ischemic stroke phase, or ISP (the last 5 min of the MCAO, that is, 25–30 min after initiation), and the immediate reperfusion phase, or IRP (the first 10 min of reperfusion). In the subacute phase, the animals were immobilized carefully for 10 min for habituation, with the EEG activity then being recorded for 5 min with the animal awake daily for 7 days. The analyses were run at frequencies of up to 40 Hz, and then split into four bands, that is, delta (1–4 Hz), theta (4–8 Hz), alpha (8–12 Hz), and beta (12–28 Hz) waves (26). The relative bandpower (qEEG of the delta, theta, alpha, and beta waves) and the qEEG indices (the delta/theta ratio [DTR], delta/alpha ratio [DAR] and [delta + theta]/[alpha + beta] or DTABR ratio) were then quantified (16, 18).

### Area of Cerebral Infarction

The rats were euthanized and perfused transcardially with phosphate-buffered saline (pH 7.4) at 4°C, followed by 4% formaldehyde (pH 7.4). The brain was extracted post-reperfusion, fixed in 4% formaldehyde for 24 h, cryoprotected in 30% sucrose for 24 h, and then cut into serial coronal sections (40 μm). The sections were stained with cresyl violet (0.3%) to measure the area (percentage) of cerebral infarction, as it offers the opportunity to test specific markers (27). These sections were mounted on gelatin-coated slides, and then immersed for 5 min at a time in a sequence of decreasing concentrations of alcohol (90, 70, and 50%). They were then placed for 5 min in 5% acetic acid solution. Following this, they were immersed in a 0.3% cresyl violet solution for 10 min and then placed in increasing alcohol solutions for 3 min each time (70, 90, and 100%). Finally, the sections were clarified and assembled in a xylol-based medium.

The brain section was chosen based on the position of the electrode in the dura mater, and photographed using a light field microscope, with the images being processed and the area of infarction being analyzed using ImageJ software (NIH, Bethesda, MD, USA). The percentage of cerebral infarction area (PIA) was calculated using the formula: PIA = (infarction area/area of the ipsilateral hemisphere) ×100.

### Statistical Analyses

The normality of the data variances was verified using the Kolmogorov-Smirnov test. All data are presented as the mean and standard deviation (SD), and the *F* and *p*-values are included, when pertinent. A *p* < 0.05 significance level was considered for all analyses. Differences between pairs of groups were analyzed using Student's *t*-test, while those among three or more groups were evaluated using a one-way or two-way Analysis of Variance (ANOVA), followed by Tukey's test for multiple comparisons. The data were analyzed using GraphPad Prism, version 8 (Graph-Pad Software Inc., San Diego, CA, USA).

## Results

### Delta Wave Bandpower and qEEG Indices Increase in the Acute Phase

**Figure 2:**
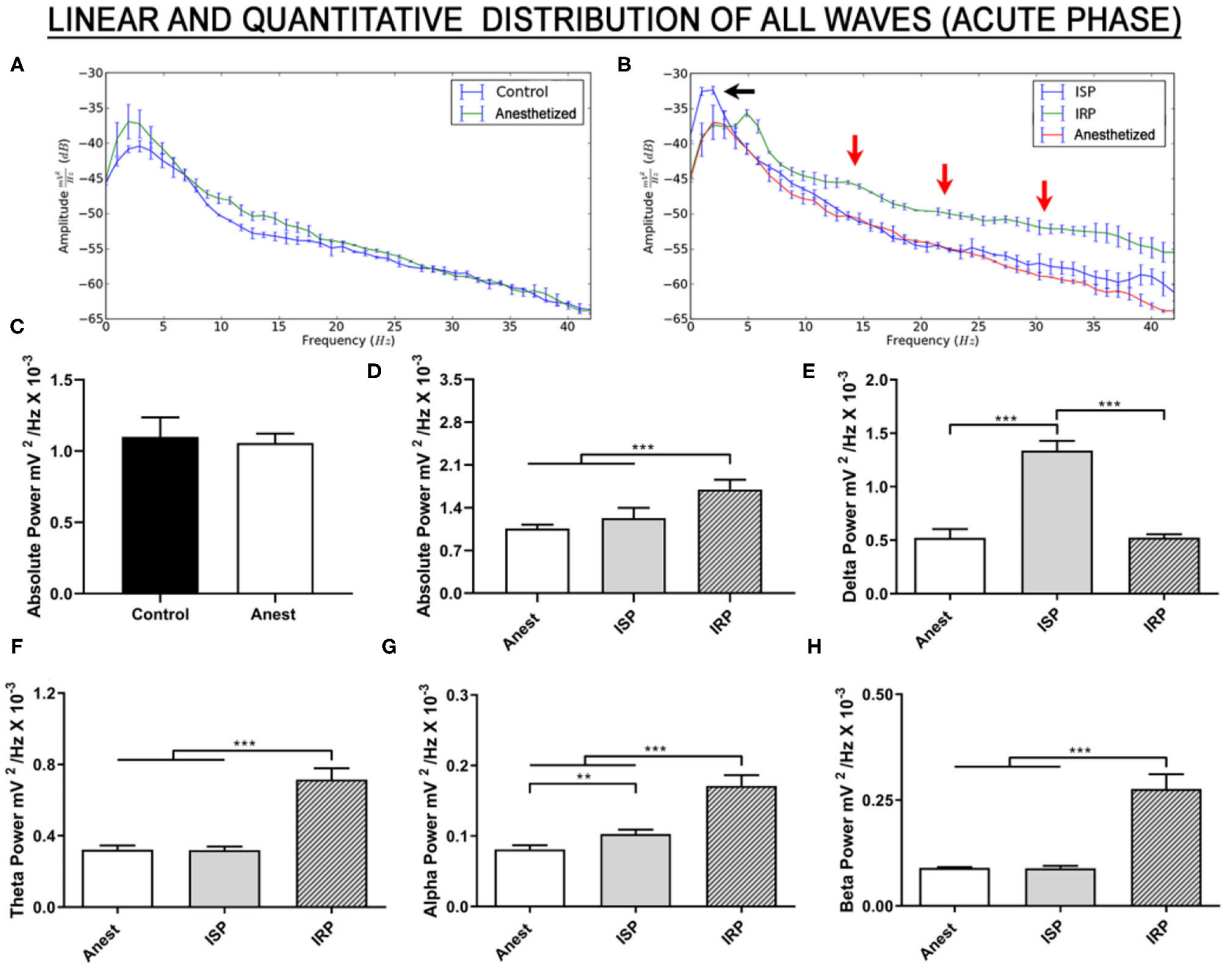
Linear frequency distribution of the animals submitted to the MCAO surgery during the acute phase. **(A)** Linear frequency distribution between the anesthetized and control (conscious) animals. **(B)** Linear frequency distribution of the anesthetized, ischemic stroke, and immediate reperfusion phase animals. **(C)** Quantitative electroencephalographic data on the absolute brainwave power of the control and anesthetized animals. **(D)** Quantitative electroencephalographic data on the absolute brainwave power, **(E)** Relative delta wave bandpower, **(F)** Relative theta wave bandpower, **(G)** Relative alpha wave bandpower, and **(H)** Relative beta wave bandpower. The data are expressed as the mean ± SD (*n* = 6 animals per group; *p < 0.05, **p < 0.01, and ***p < 0.0001). Anest, anesthetized animals; ISP, ischemic stroke phase; IRP, immediate reperfusion phase.

Brainwaves can help to identify ischemic damage in the acute phase of a stroke. Here, the absolute brainwave power of the anesthetized animals was not statistically different from that of the control (conscious) animals (Figures 2A,C, Supplementary Table 1; *p* = 0.4916). Oscillations were recorded in the low-frequency waves during the MCAO (Figure 2B, black arrow) and in the high-frequency waves at the onset of reperfusion (Figure 2B, red arrows).

In the case of the absolute brainwave power up to 40 Hz [*F*_(2, 15)_ = 33.98; *p* < 0.0001; Figure 2D, Supplementary Table 1], no significant variation was found between the ischemic stroke phase (ISP) and the anesthetized group (*p* = 0.1220). In the immediate return of blood flow (IRP), however, absolute brainwave power increased significantly in comparison with both the anesthetized and ISP groups (*p* < 0.0001, for both comparisons).

The brainwaves (relative bandpower) were also decomposed. There was a significant increase in delta waves during ischemia [*F*_(2, 15)_ = 247.1, *p* < 0.0001; vs. anesthetized: *p* < 0.0001; Figure 2E, Supplementary Table 1], although the relative delta bandpower during the immediate reperfusion returned to values similar to those recorded in the anesthetized group (ischemia vs. anesthetized: *p* = 0.9984; vs. ISP: *p* < 0.0001). This indicates intense delta wave activity during the MCAO, followed by a reduction in this activity with the immediate return of the blood flow.

By contrast, no significant alteration was observed in the relative bandpower of the theta waves during the ischemic event [*F*_(2, 15)_ = 190.2, *p* < 0.0001; vs. anesthetized: *p* = 0.9917; Figure 2F, Supplementary Table 1]. A significant increase was nevertheless observed in the first 10 min of the return of blood flow (vs. anesthetized: *p* < 0.0001; and vs. ISP: *p* < 0.0001).

All the groups were significantly different from each another in their alpha wave frequencies [*F*_(2, 15)_ = 130.2; *p* < 0.0001], with a significant increase in both the ISP (vs. anesthetized: *p* = 0.0054) and IRP (vs. anesthetized: *p* < 0.0001) groups in comparison with the anesthetized group, reflecting the significantly greater alpha wave activity during the reperfusion phase in comparison with the ischemic phase (*p* < 0.0001; Figure 2G, Supplementary Table 1).

As observed in the case of the theta waves, the relative bandpower of the beta waves only increased significantly following the onset of the return of the blood flow to the brain [*F*_(2, 15)_ = 167.7, *p* < 0.0001; vs. anesthetized: *p* < 0.0001; and vs. ISP: *p* < 0.0001; Figure 2H, Supplementary Table 1). This indicates disorderly electrical activity in the rhythm of the beta waves during the early reperfusion. No difference was found between the anesthetized and ISP groups (*p* = 0.9956).

Taken together, these findings indicate that, during cerebral ischemia, there is an increase in the relative delta wave bandpower, and that, during the immediate reperfusion, there is an increase in all the other brainwaves. These findings may be crucial to clinical decision-making.

The qEEG indices (DTR, DAR, and DTABR) were used to predict and monitor the evolution of the stroke in the acute phase. During the ischemic stroke phase, the values of all the indices were 2–3 times higher than those recorded in the anesthetized animals, although this difference was reduced during the reperfusion phase (Table 1). Taken together, these indices may provide insights into the cerebral damage cause by acute ischemic events, and in particular, guidelines for the clinical decision-making necessary to implement successful reperfusion therapy.

**Table 1:** Quantitative EEG indexes to predict and monitor the evolution of stroke in the acute phase.

	**DAR**	**DTABR**	**DTR**
**Anesthetized**			
Mean	6.45	4.95	1.62
SD	± 1.21	± 0.68	± 0.21
**ISP**			
Mean	13.07^a^	8.68^a^	4.20^a^
SD	± 1.48	± 0.91	± 0.36
**IRP**			
Mean	3.08^a, b^	2.80^a, b^	0.74^a, b^
SD	± 0.27	± 0.34	± 0.10
*p < 0.05*	a = vs. anesthesia b = vs. ISP		
*F*-value	123.8	111.5	308.5

*a = vs. anesthesia; b = vs. ISP. The data are expressed as mean ± SD (n = 6 animals/group)*.

### An Important Window of Change in Electrical Activity in the Brain Between D3 and D5 May Be Critical for the Post-Ischemic Recovery Period

**Figure 3:**
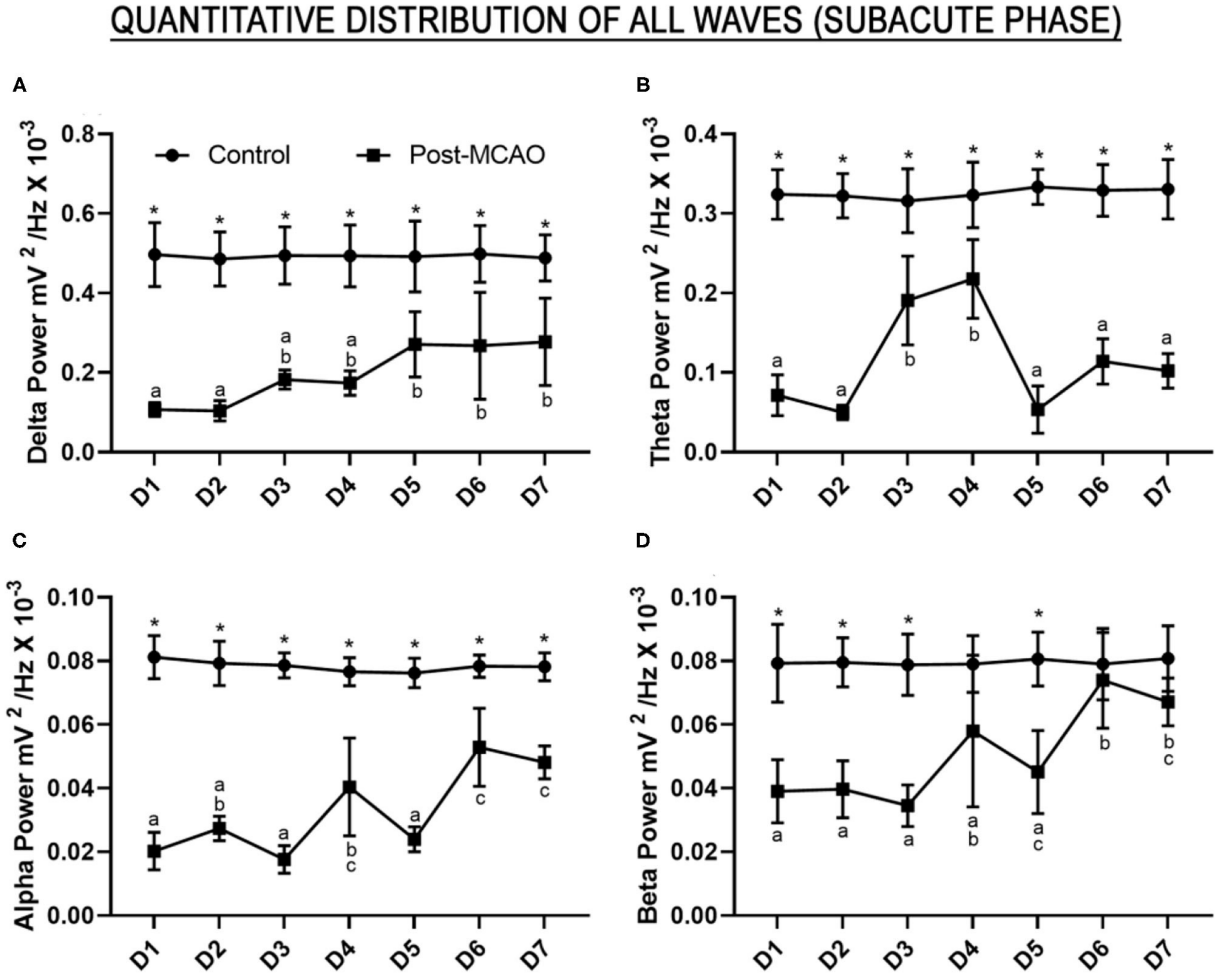
Relative bandpower of all the brainwaves (1–28 Hz) of the post-ischemic animals monitored for 7 days. **(A)** Quantitative electroencephalographic data on the relative delta wave bandpower; **(B)** Relative theta wave bandpower; **(C)** Relative alpha wave bandpower; **(D)** Relative beta wave bandpower. The data are expressed as means±SD (*n* = 6 animals per group); *p < 0.05: control vs. post-MCAO group. Different letters above the data points denote significant differences between days (*p* < 0.05).

A number of studies have indicated that the continuous monitoring of relative brainwave bandpower, and the DAR, DTR, and DTABR indices may provide important insights into the efficacy of reperfusion therapies or the use of neuroprotection and neurorepair drugs in an experimental approach. In the present study, the relative bandpower of the delta waves was lower than that of the control group during all 7 days of the reperfusion period [*F*_(6, 70)_ = 3.060, *p* = 0.0102; D1–D7 vs. control, *p* < 0.001; Figure 3A, Supplementary Table 2]. We also recorded a gradual increase in the frequency of the delta waves between the beginning and end of reperfusion, with significant differences being recorded from D5 onward (D1 vs. D5–D7: *p* < 0.05; D2 vs. D5–D7: *p* < 0.05). This indicates a partial recovery of electrical activity in this brainwave.

Although the theta wave activity was lower than that recorded in the control animals during the continuous monitoring [*F*_(6, 70)_ = 12.37, *p* < 0.0001; control vs. all post-MCAO days: *p* < 0.0001], there was an increase in the firing-rate amplitude on days D3 and D4 in comparison with the other days (D3 vs. D1, D2, D5, D6, and D7: *p* < 0.05; D4 vs. D1, D2, D5, D6, and D7: *p* < 0.001; D3 vs. D4: *p* = 0.9830; Figure 3B, Supplementary Table 2). This may reflect an important window of change in electrical brain activity on these 2 days.

In the case of the high-frequency waves, the continuous analysis of the alpha waves also indicated that the bandpower was lower than that recorded in the control group [*F*_(6, 70)_ = 13.33, *p* < 0.0001; control vs. all post-MCAO days: *p* < 0.0001, Figure 3C, Supplementary Table 2]. An increase in the relative bandpower of the alpha waves was also observed on days D4, D6, and D7 in comparison with the other days (D4 vs. D1, D3, and D5: *p* < 0.01; D4 vs. D6 and D7: *p* > 0.05; D6 vs. D1, D2, D3, and D5: *p* < 0.0001; D7 vs. D1, D2, D3, and D5: *p* < 0.001; D6 vs. D7: *p* = 0.9955). This indicates disturbances in the electrical activity of the alpha waves during tissue reperfusion.

The qEEG of the animals submitted to MCAO presented beta wave activity significantly lower than that observed in the control group on days D1–D3 and D5 [*F*_(6, 70)_ = 5.064, *p* = 0.0002; D1, D2, D3, and D5 vs. control, *p* < 0.001; Figure 3D, Supplementary Table 2], although no significant variation was observed on days D4, D6, or D7 (control vs. D4: *p* = 0.1263; vs. D6: *p* > 0.9999; and vs. D7: *p* = 0.7525). The beta wave activity recorded on D6 was also significantly different from D1 (*p* = 0.0002), D2 (*p* = 0.0003), D3 (*p* < 0.0001), and D5 (*p* = 0.0045), while D7 was significantly different from D1 (*p* = 0.0065), D2 (*p* = 0.0088), and D3 (*p* = 0.0006), which indicates the recuperation of beta wave electrical activity to normal levels.

**Figure 4:**
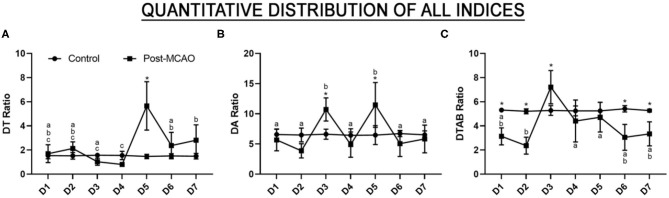
Quantitative EEG predictor index and the results of the monitoring of the evolution of the stroke in the subacute phase. **(A)** Quantitative electroencephalographic data on the delta/theta ratio; **(B)** The delta/alpha ratio; **(C)** The delta+theta/alpha+beta ratio. The data are expressed as the means ± SD (*n* = 6 animals per group); *p < 0.05: control vs. post-MCAO group. Different letters above the data points denote significant differences between days (*p* < 0.05).

A significant increase was observed in the DTR on day D5 in comparison with the control group [*F*_(6, 70)_ = 14.06, *p* < 0.0001; control vs. D5, *p* < 0.0001; Figure 4A, Supplementary Table 2], which was probably caused by a decrease in the theta wave on this day. The DAR presented a similar pattern to the DTR on D5, with a second peak on D3, in comparison with the control group [*F*_(6, 70)_ = 8.862, *p* < 0.0001; control vs. D3, *p* = 0.0095; and vs. D5, *p* = 0.0004; Figure 4B, Supplementary Table 3].

Interestingly, the DTABR values recorded on the first 2 days were significantly lower than those recorded in the control group [*F*_(6, 70)_ = 10.58, *p* < 0.0001; control vs. D1: *p* = 0.0034; vs. D2: *p* < 0.0001; Figure 4C, Supplementary Table 3] and were followed by a major increase on D3 (vs. D3, *p* = 0.0165). This peak was followed by a gradual reduction to levels similar to that of the control group by D4 (control vs. D4, *p* = 0.9181) and D5 (control vs. D5, *p* = 0.9987), with significantly lower values being recorded on D6 (control vs. D6, *p* > 0.0009) and D7 (control vs. D7, *p* = 0.0172). These findings indicate that there is a prominent window of electrical brain disorder between D3 and D5, which may be critical for the pathophysiology of ischemia and reperfusion.

**Figure 5:**
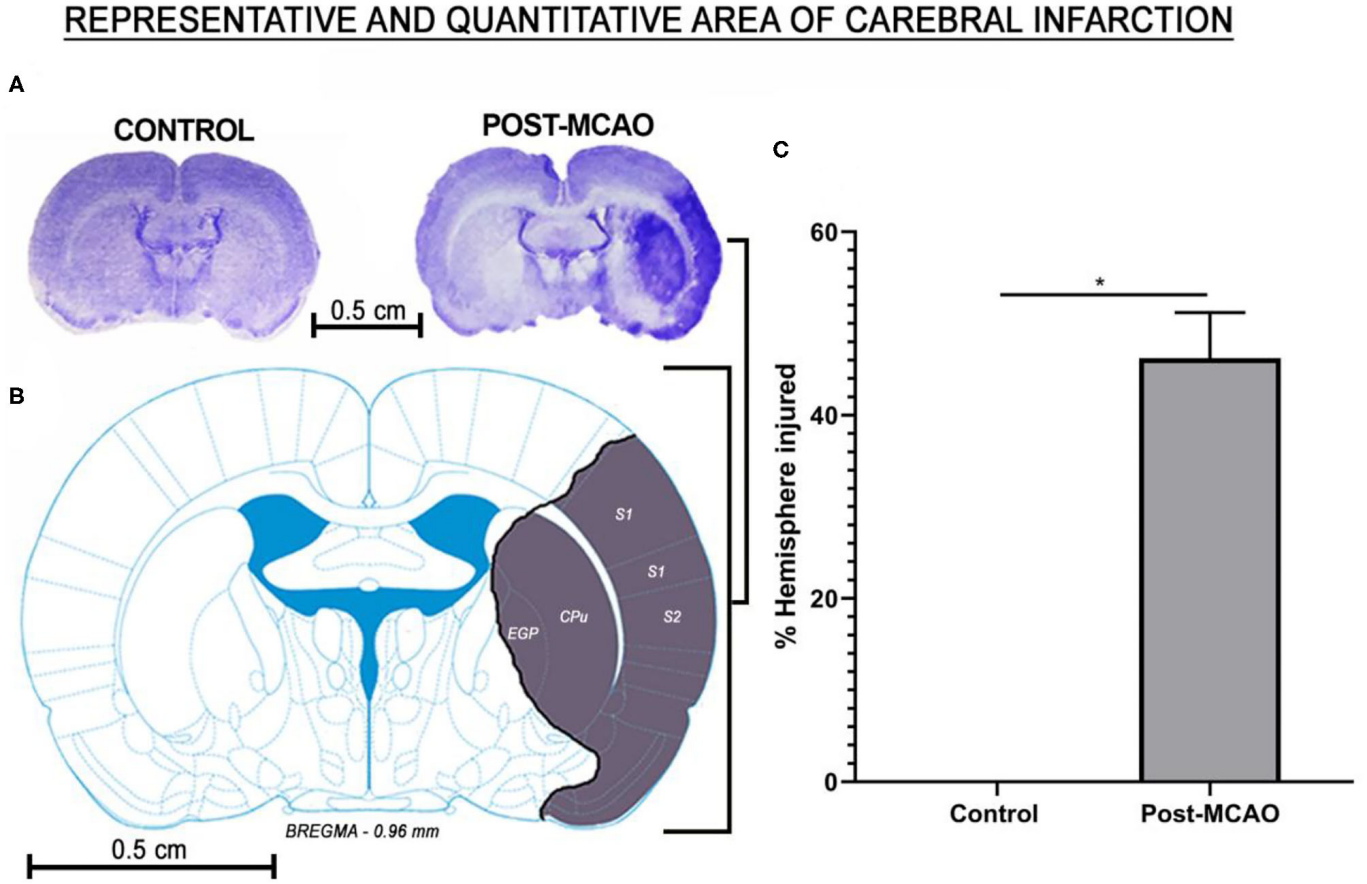
Affected area and the percentage of cerebral infarction area. **(A)** Representative image of the injured hemisphere in which the electrodes were implanted: control (left) and post-MCAO (right). **(B)** Schematic representation of the injured regions. **(C)** Mean percentage of injured cerebral hemisphere. The data are expressed as means ± SD (*n* = 6 animals per group); *p < 0.05: control vs. post-MCAO group. S1, primary somatosensory area; S2, secondary somatosensory area; EGP, external globus pallidus; CPu, caudate putamen.

The electrical changes recorded in the ischemic stroke phase, which were highly suggestive of ischemic injury, were confirmed at the end of the experiment (after 7 days of reperfusion). In other words, the animals submitted to cerebral ischemia presented cortical-subcortical injuries with damage in the primary and secondary somatosensory areas, the caudate putamen, and the external globus pallidus (Figures 5A,B, Supplementary Figure 1). A mean of 46.22 ± 4.99% of the hemisphere was lesioned (Figure 5C).

## Discussion

In the acute phase of the present study, we recorded a marked increase in the relative delta wave bandpower and a smaller, but significant increase in the relative alpha wave bandpower in the ischemic stroke phase (ISP). In the immediate reperfusion phase, however, we observed an increase in the theta, alpha, and beta wave bandpower, but no alteration in the delta wave activity, in comparison with the anesthetized group. In the subacute phase, however, all wave types remained lower than those of the control group, while the DTR, DAR, and DTABR indices increased relative to the control, indicating high rates of brain electrical activity between D3 and D5 in the post-ischemic recovery period.

We observed distinct patterns of the distribution of delta wave activity between the ischemia and reperfusion periods. The delta rhythms also peaked during the blockage of the blood flow (ischemic stroke phase), which may reflect a high level of neuronal damage by hypoxia (28). Delta wave activity is believed to originate in the thalamic neurons and the deep cortical layers, which may represent the sustained hyperpolarization and inhibition of the injured cortical neurons (29). In this respect, our findings are consistent with those of previous studies, in which a major increase in delta wave activity, when associated with motor disability, is often linked to acute brain damage, lasting from minutes to days (2, 16, 18), which is compatible with a stroke in the cortical-subcortical regions (28).

It is interesting to note that the blood flow has a direct relationship with delta wave activity. Foreman and Claassen (17) showed that abnormal EEG activity begins to appear as soon as the blood flow decreases, and when it exceeds the ischemic threshold of 18 ml/100 g/min, there is an increase in delta wave activity, which is perceived by the electrode. This implies that qEEG is a reliable method for the assessment of acute ischemic brain injury (30). Although only a single pair of electrodes was used in the present study, which may have restricted its spatial accuracy in comparison with systems that employ multiple electrodes, Johnstone et al. (31) and Hemington and Reynolds (32) both validated this approach for EEG recording and diagnosis. One other limitation of the study is the lack of any possibility of evaluating other clinical features of the acute phase of ischemic stroke, such as aphasia, mental confusion or visual impairment.

An important novel finding of the present study is the abrupt reduction in delta wave activity to control levels at the moment of return of the blood flow. Even though few studies have described this phenomenon (20), the recuperation of delta wave activity to normal levels during reperfusion may reflect the efficiency of reperfusion therapy based on thrombolytic drugs, and may thus be used as an indicator of successful treatment or for the future evaluation of the effectiveness of neurorepair drugs that improve brain wave patterns.

The alpha wave is emitted by thalamocortical interactions through the nucleus reticularis (29) and regulates signaling within the cortex (33). In the specific case of the relative alpha wave bandpower, a number of studies have demonstrated a relationship with optimal cerebral arousal (34, 35). Previous studies (2, 36) have reported that the preservation or an increase in alpha wave activity in stroke patients indicates the survival of neurons in areas affected by blocked blood flow. This is consistent with our findings of an increase in alpha wave activity during ischemic stroke and an even greater increase during the immediate reperfusion phase, which predicts a potentially positive effect of reperfusion therapy.

In addition to these findings, the bandpower of the theta and beta waves did not change in the ischemic stroke phase, but it did increase significantly in the immediate reperfusion phase. The identification of the physiological origin of these rhythms is still open to debate, although a number of studies have shown that the theta waves may originate from projections of the nucleus basalis, cingulate gyrus, and thalamus to the cortical areas (29). These waves are considered to be an unreliable index of stroke pathophysiology, when considered in isolation, as they may be confounded by reduced alpha wave activity (16), although they do appear to be sensitive to variations in blood flow, primarily in the context of reperfusion (12, 37, 38). In the present study, we recorded normal theta wave activity in the EEGs recorded during the ischemia, which contrasts with the pattern found in previous studies (16, 18). Although the beta waves may reflect the cortico-cortical and thalamocortical projections (39), they are contaminated by electromyographic interference (39), and are this considered to be an unreliable index (39), with no relevant higher-order output (5, 16).

Different wave patterns in the two acute phases (ischemia and reperfusion) were reflected in different indices. In particular, we recorded high values for the DTR, DAR, and DTABR indices during the ischemia, followed by a major reduction during reperfusion. Aminov et al. (18) recorded a similar pattern in the case of the DTR, and associated an increase in the DTR with a poor cognitive outcome. Although the DTR is a predictive index of functional or cognitive outcomes (12, 13), few studies have focused specifically on this aspect of the process (18), and further research is required to provide more systematic insights into the association between this EEG metric and the cognitive outcome.

Finnigan et al. (5, 16) and other authors (18, 28) also reported an increase in the DAR during ischemia and in ischemic stroke patients within days of the onset of the symptoms. In particular, Finnigan et al. (16) observed a 1:1 (sensitivity:specificity) ratio for the DAR in the discrimination of acute ischemic stroke, with a threshold of 3.7. Interestingly, we found supra-threshold values at the exact moment of the ischemia, followed by a major reduction in reperfusion, which indicates that the DAR may also be an appropriate index for the assessment of the process, assuming that the reperfusion therapy were effective. Schleiger et al. (40) found a significant improvement in the EEG parameters following thrombectomy, with a gradual reduction in the DAR after the procedure. It is important to note that a reduction in the DAR is a response to the return of blood flow, which is not always associated with tissue recovery or restored brain function (40). The present study is thus one of the first to demonstrate a reduction in the DAR in the immediate reperfusion phase.

In this context, Finnigan et al. (16) and Sheorajpanday et al. (6) proposed that the DTABR had a sensitivity of 100% for ischemic injury above 3.5, which is consistent with our findings, given that we recorded a value much higher than the proposed threshold. Even so, this index is less specific than the DAR, given the interference from the beta and theta waves.

In the present study, we provide a subacute assessment of the brainwaves and the derived indices in animals submitted to cerebral ischemia followed by reperfusion for 7 days. We recorded delta, theta, alpha, and beta wave activity at levels below those recorded in the control group during the study period. However, we did observe a tendency for a return to baseline levels from D4 (alpha and beta waves) and D5 (delta wave) onward. Lu et al. (41) obtained similar results, in particular, a partial return to baseline levels in the case of the delta waves. Yin et al. (30) considered the delta wave bandpower to be one of the most important qEEG metrics for prognosis, together with the alpha waves and the DAR index.

In the specific case of the theta and alpha waves, in addition, we observed values well below those presented by the control group throughout the seven days of the study period, although we did record a marked increase in the activity of these waves on D3 and D4. Lu et al. (41) recorded a similar increase in theta wave activity after 72 h of reperfusion, with a subsequent stabilization, which contrasts with the findings of the present study, given that theta wave activity decreased after D4, indicating a possible failure in the repair mechanisms. We would interpret this as a possible attempt to recuperate tissue or function, given that both theta and alpha waves are related to proper brain function (35) or a reflection of the glial activation of the microglia and astrocytes (42), which begins to increase from the third day after ischemia and may alter the electrical mechanisms of signal transduction (43).

The present study also provides a novel approach for the assessment of the DTR, DAR, and DTABR indices, based on a daily subacute diagnosis. These indices may thus provide an extremely valuable tool for the diagnosis of post-stroke pathophysiology, although few studies have focused specifically on these indices as a prognostic factor following surgical or pharmacological interventions through the continuous monitoring of post-onset reperfusion in patients with ischemic stroke (20, 40). In this context, it is important to note that the return of blood flow (oxygen and glucose) induces transient electrical changes before restoring baseline conditions, which may reflect reperfusion injury (44, 45). These changes are present during the first week and up to 1 month after the initiation of tissue recovery, and thus play an important role in the neuroplasticity following a stroke (44, 46). We would suggest that these changes occur between D3 and D5, during the first week, and that the increase in the DAR and DTR indices on these days may be related to the reorganizations of cells in the brain following the ischemia. This would support the application of neuroreparative therapies in experimental models for at least up to fifth day. Obviously, further research on a broader scale or focusing on multiple neuronal centers would be necessary to confirm the effectiveness of these indices for the evaluation of clinical improvement, beginning with reperfusion.

In addition, some studies have shown the relationship between qEEG and brain function after stroke. Finnigan et al. (47) evaluated the neurological function using the National Institutes of Health Stroke Scale (NIHSS) in patients at admission and 30 das after the stroke and found a strong correlation between delta waves and NHISS scores, where the lower the delta index, the lower the NHISS scores. Moreover, Sheikh et al. (48) and Schleiger et al. (40) reported patients who were admitted with NIHSS > 15 and abnormal DAR, who after receiving reperfusion therapy improved neurological function and reduced DAR and symptoms, such as aphasia, forced gaze deviation, and hemiparesis.

Overall, then, the present study has shown that the early and accurate identification of decreasing delta wave bandpower, and DTR, DAR, and DTABR indices, together with an increase in the bandpower of the other waves in the immediate reperfusion phase may represent a major advance in our capacity to evaluate the effectiveness of reperfusion therapy. The study also indicated that a single pair of electrodes can provide valuable diagnostic parameters, and can be connected to mobile devices, thereby improving screening and decision-making on patients who may have been affected by ischemic brain injury.

## Data Availability Statement

The raw data supporting the conclusions of this article will be made available by the authors, without undue reservation.

## Ethics Statement

The animal study was reviewed and approved by Ethics Committee on Use of Animals of the Biological Sciences Institute at the Federal University of Pará.

## Author Contributions

LF and BM: performed the protocol and drafted the manuscript. VJ, EY, and EC: conducted the bioinformatic analysis and interpreted the results. AM-F: analyzed the data and drafted the manuscript. MH and DL: reviewed and edited the manuscript. All authors: contributed to manuscript revision, read and approved the submitted version.

## Conflict of Interest

The authors declare that the research was conducted in the absence of any commercial or financial relationships that could be construed as a potential conflict of interest.
