# Supplementary material for: Increased Relative Delta Bandpower and Delta Indices Revealed by Continuous qEEG Monitoring in a Rat Model of Ischemia-Reperfusion
Source: Front Neurol. 2021 Apr 7;12:645138. doi: 10.3389/fneur.2021.645138 (PMC8058376; doi:10.3389/fneur.2021.645138)
Supplement: Supplementary file 1 [file Data_Sheet_1.docx]

*Supplementary Material*

ST1 (Supplementary Table 1) – Quantitative electroencephalographic data on the absolute and decomposition brainwave in acute phase.

|  | Mean | Std. Deviation |
| --- | --- | --- |
| Absolute Power |  |  |
| *Control* | 1.101 | ± 0.135 |
| *Anest* | 1.057 | ± 0.065 |
|  |  |  |
| Absolute Power |  |  |
| *Anest* | 1.057 | ± 0.065 |
| *ISP* | 1.227 | ± 0.168 |
| *IRP* | 1.697^*#^ | ± 0.159 |
|  |  |  |
| Delta Power |  |  |
| *Anest* | 0.522 | ± 0.081 |
| *ISP* | 1.338^*^ | ± 0.091 |
| *IRP* | 0.524^#^ | ± 0.030 |
|  |  |  |
| Theta Power |  |  |
| *Anest* | 0.322 | ± 0.023 |
| *ISP* | 0.319 | ± 0.020 |
| *IRP* | 0.715^*#^ | ± 0.062 |
|  |  |  |
| Alpha Power |  |  |
| *Anest* | 0.081 | ± 0.005 |
| *ISP* | 0.102^*^ | ± 0.006 |
| *IRP* | 0.171^*#^ | ± 0.015 |
|  |  |  |
| Beta Power |  |  |
| *Anest* | 0.089 | ± 0.002 |
| *ISP* | 0.088 | ± 0.006 |
| *IRP* | 0.276^*#^ | ± 0.034 |

Data are presented as means ± SD. **p* < 0.05 vs Anest; ^#^*p* < 0.05 vs ISP. Anest: anesthetized animals; ISP: ischemic stroke phase; and IRP: immediate reperfusion phase.

ST2 (Supplementary Table 2) – Relative bandpower of all the brainwaves of the post-ischemic animals during 7 days.

|  | **Delta Wave** | | | |
| --- | --- | --- | --- | --- |
|  | Control | | Post-MCAO | |
| Days | *Mean* | *Std. Deviation* | *Mean* | *Std. Deviation* |
| *D1* | 0.497 | ± 0.081 | 0.107^*a^ | ± 0.017 |
| *D2* | 0.486 | ± 0.068 | 0.104^*a^ | ± 0.026 |
| *D3* | 0.494 | ± 0.072 | 0.183^*ab^ | ± 0.024 |
| *D4* | 0.493 | ± 0.078 | 0.174^*ab^ | ± 0.031 |
| *D5* | 0.492 | ± 0.089 | 0.271^*b^ | ± 0.082 |
| *D6* | 0.498 | ± 0.071 | 0.268^*b^ | ± 0.134 |
| *D7* | 0.488 | ± 0.058 | 0.277^*b^ | ± 0.109 |
|  |  |  |  |  |
|  | **Theta Wave** | | | |
|  | Control | | Post-MCAO | |
|  | Mean | Std. Deviation | Mean | Std. Deviation |
| *D1* | 0.324 | ± 0.031 | 0.072^*a^ | ± 0.026 |
| *D2* | 0.322 | ± 0.028 | 0.050^*a^ | ± 0.009 |
| *D3* | 0.316 | ± 0.040 | 0.191^*b^ | ± 0.056 |
| *D4* | 0.323 | ± 0.041 | 0.218^*b^ | ± 0.050 |
| *D5* | 0.334 | ± 0.022 | 0.053^*a^ | ± 0.030 |
| *D6* | 0.329 | ± 0.032 | 0.114^*a^ | ± 0.029 |
| *D7* | 0.330 | ± 0.037 | 0.102^*a^ | ± 0.022 |
|  |  |  |  |  |
|  | **Alpha Wave** | | | |
|  | Control | | Post-MCAO | |
|  | Mean | Std. Deviation | Mean | Std. Deviation |
| *D1* | 0.081 | ± 0.007 | 0.020^*a^ | ± 0.006 |
| *D2* | 0.079 | ± 0.007 | 0.027^*ab^ | ± 0.004 |
| *D3* | 0.079 | ± 0.004 | 0.018^*a^ | ± 0.004 |
| *D4* | 0.077 | ± 0.004 | 0.040^*c^ | ± 0.015 |
| *D5* | 0.076 | ± 0.005 | 0.024^*a^ | ± 0.004 |
| *D6* | 0.078 | ± 0.004 | 0.053^*c^ | ± 0.012 |
| *D7* | 0.078 | ± 0.004 | 0.048^*c^ | ± 0.005 |
|  |  |  |  |  |
|  | **Bate Wave** | | | |
|  | Control | | Post-MCAO | |
|  | Mean | Std. Deviation | Mean | Std. Deviation |
| *D1* | 0.079 | ± 0.012 | 0.039^*a^ | ± 0.010 |
| *D2* | 0.080 | ± 0.008 | 0.040^*a^ | ± 0.009 |
| *D3* | 0.079 | ± 0.010 | 0.035^*a^ | ± 0.007 |
| *D4* | 0.079 | ± 0.009 | 0.058^ab^ | ± 0.024 |
| *D5* | 0.081 | ± 0.008 | 0.045^*ac^ | ± 0.013 |
| *D6* | 0.079 | ± 0.011 | 0.074^b^ | ± 0.015 |
| *D7* | 0.081 | ± 0.010 | 0.067^bc^ | ± 0.007 |

Data are presented as means ± SD. *p < 0.05: control vs post-MCAO group. Different letters above the data points denote significant differences between days (p < 0.05). MCAO: middle cerebral artery occlusion.

ST3 (Supplementary Table 3) – Quantitative EEG predictor index and the results of the evolution of the stroke in the subacute phase.

|  | **DTR** | | | |
| --- | --- | --- | --- | --- |
|  | Control | | Post-MCAO | |
| Days | *Mean* | *Std. Deviation* | *Mean* | *Std. Deviation* |
| *D1* | 1.541 | ± 0.263 | 1.698^abc^ | ± 0.739 |
| *D2* | 1.518 | ± 0.269 | 2.130^abc^ | ± 0.551 |
| *D3* | 1.572 | ± 0.187 | 1.028^ac^ | ± 0.294 |
| *D4* | 1.551 | ± 0.346 | 0.805^c^ | ± 0.054 |
| *D5* | 1.467 | ± 0.180 | 5.656^*^ | ± 2.003 |
| *D6* | 1.516 | ± 0.182 | 2.374^ab^ | ± 1.091 |
| *D7* | 1.491 | ± 0.227 | 2.814^b^ | ± 1.270 |
|  |  |  |  |  |
|  | **DAR** | | | |
|  | Control | | Post-MCAO | |
|  | Mean | Std. Deviation | Mean | Std. Deviation |
| *D1* | 6.577 | ± 0.858 | 5.678^a^ | ± 1.808 |
| *D2* | 6.498 | ± 1.140 | 3.868^a^ | ± 1.172 |
| *D3* | 6.628 | ± 0.833 | 11.723^*b^ | ± 1.915 |
| *D4* | 6.443 | ± 1.086 | 4.902^a^ | ± 2.086 |
| *D5* | 6.468 | ± 1.565 | 11.476^*b^ | ± 3.721 |
| *D6* | 6.726 | ± 0.508 | 5.051^a^ | ± 2.127 |
| *D7* | 6.527 | ± 0.645 | 5.834^a^ | ± 2.292 |
|  |  |  |  |  |
|  | **DTABR** | | | |
|  | Control | | Post-MCAO | |
|  | Mean | Std. Deviation | Mean | Std. Deviation |
| *D1* | 5.309 | ± 0.165 | 3.133^*ab^ | ± 0.706 |
| *D2* | 5.212 | ± 0.205 | 2.365^*b^ | ± 0.700 |
| *D3* | 5.278 | ± 0.404 | 7.211^*^ | ± 1.379 |
| *D4* | 5.245 | ± 0.408 | 4.406^a^ | ± 1.736 |
| *D5* | 5.243 | ± 0.708 | 4.725^a^ | ± 1.233 |
| *D6* | 5.418 | ± 0.262 | 3.053^*ab^ | ± 1.071 |
| *D7* | 5.267 | ± 0.122 | 3.341^*ab^ | ± 0.997 |

Data are presented as means ± SD. DTR: delta/theta ratio; DAR: delta/alpha ratio; DTABR: delta+theta/alpha+beta ratio. *p < 0.05: control vs post-MCAO group. Different letters above the data points denote significant differences between days (p < 0.05). MCAO: middle cerebral artery occlusion.

We performed a brief qualitative analysis of the ischemic region in the MCAO group and its corresponding location in the *sham* group (control), on an experimental area of 50 x 50 μm^2^. We characterized intact pyramidal neurons-like cells by the triangular cell body and condensed nuclei; and damaged neurons-like cells presented dark cytoplasm and fragmented nuclei spread on the whole cell. The *sham* group showed the predominance of pyramidal neurons-like with intact morphology, while in lesion area was identified cells with altered morphology.


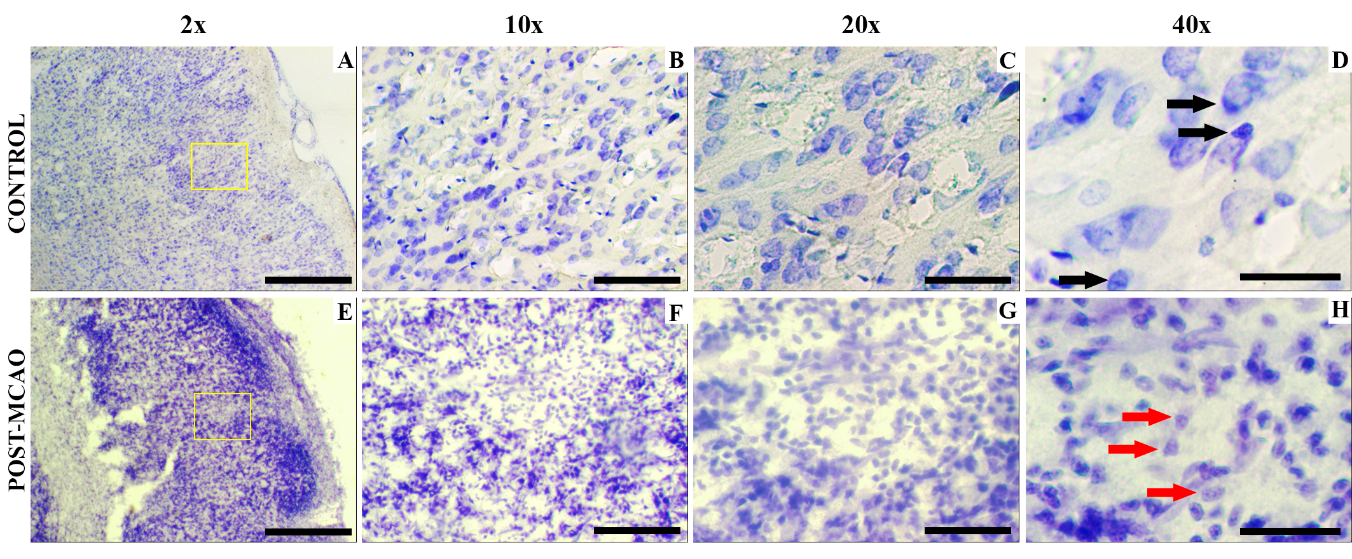


SF1 (Supplementary Figure 1) – Photomicrography of cresyl violet staining in the brain rats submitted or not to MCAO. (A-D) Animals controls (*sham* group). (E-H) MCAO group. Black arrows: intact neurons-like cells. Red arrows: damaged neurons-like cells. A and E: scale of 500 μm. B and F: scale of 100 μm (amplification of yellow square). C and G: scale of 50 μm. D and H: scale of 30 μm. MCAO: middle cerebral artery occlusion.
